# Supplementary figures and images for: Reference values of normal fetal ductus venosus Doppler flow measurements at 11–14 weeks of gestation
Source: PLoS One. 2024 Oct 28;19(10):e0312874. doi: 10.1371/journal.pone.0312874 (PMC11516001; doi:10.1371/journal.pone.0312874)

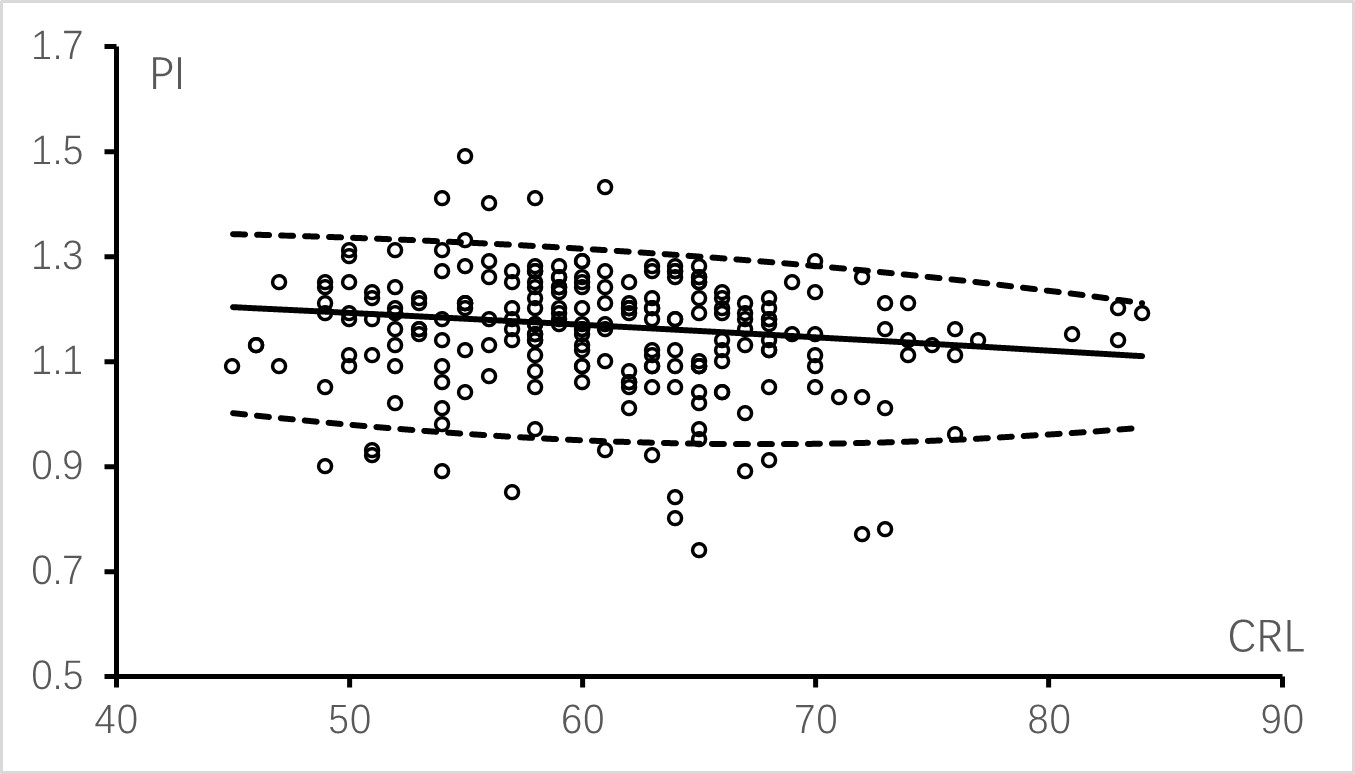

Supplement: S1 Fig — (TIF) [file pone.0312874.s001.tif]

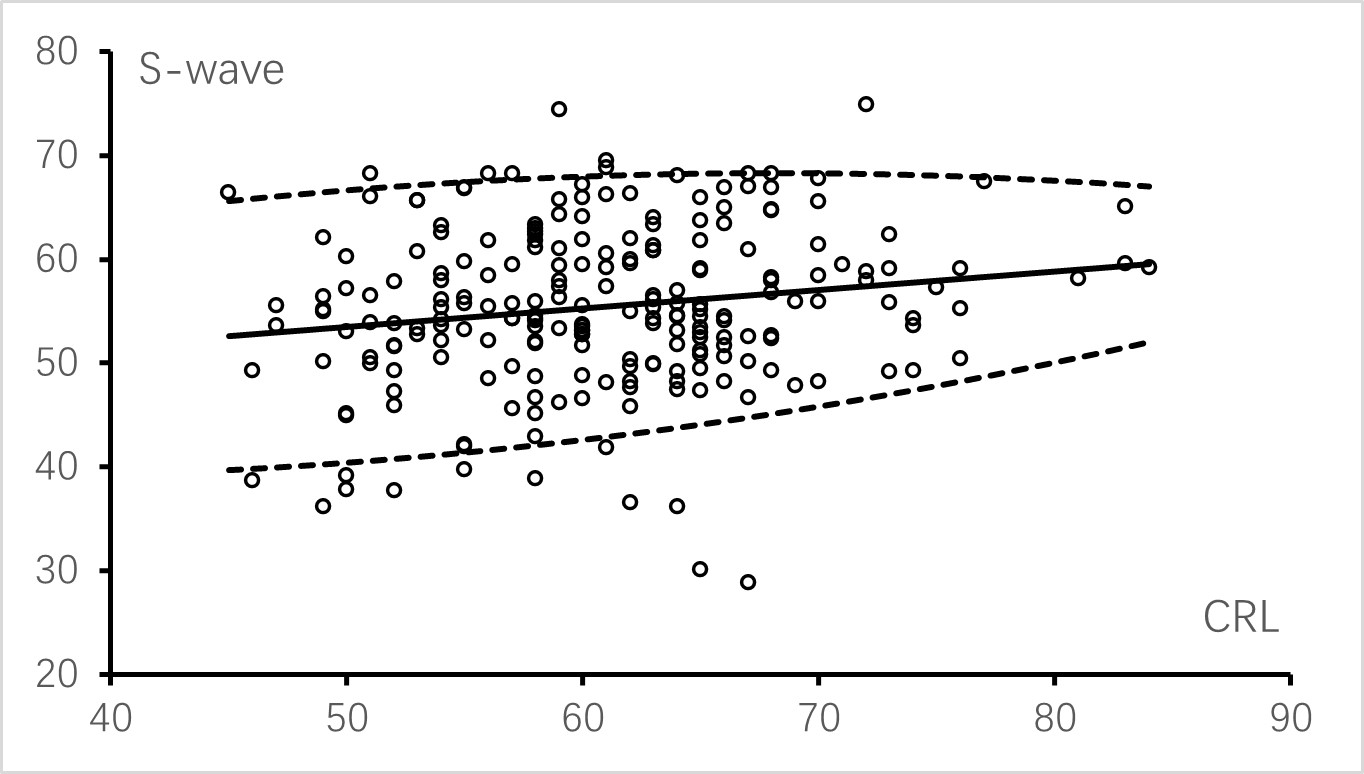

Supplement: S2 Fig — (TIF) [file pone.0312874.s002.tif]

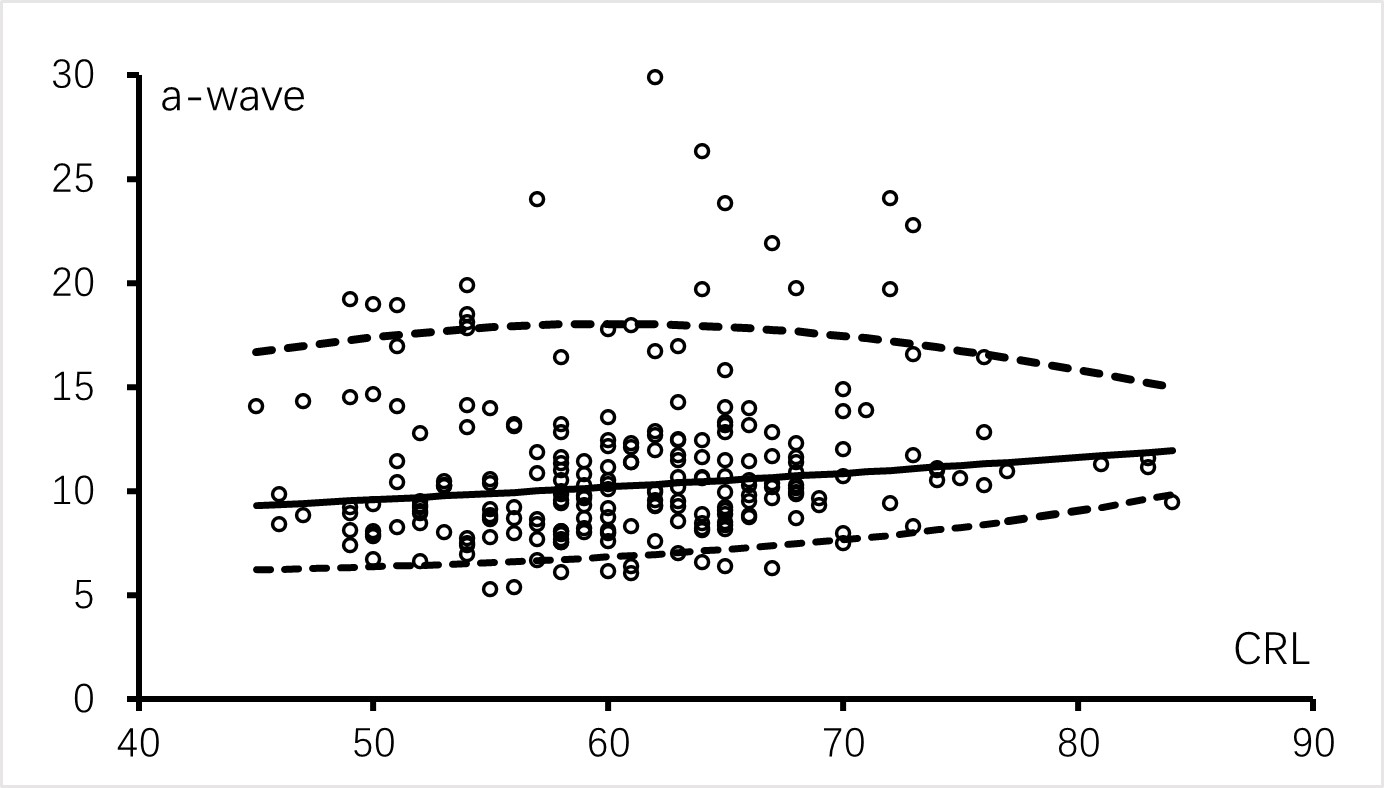

Supplement: S3 Fig — (TIF) [file pone.0312874.s003.tif]

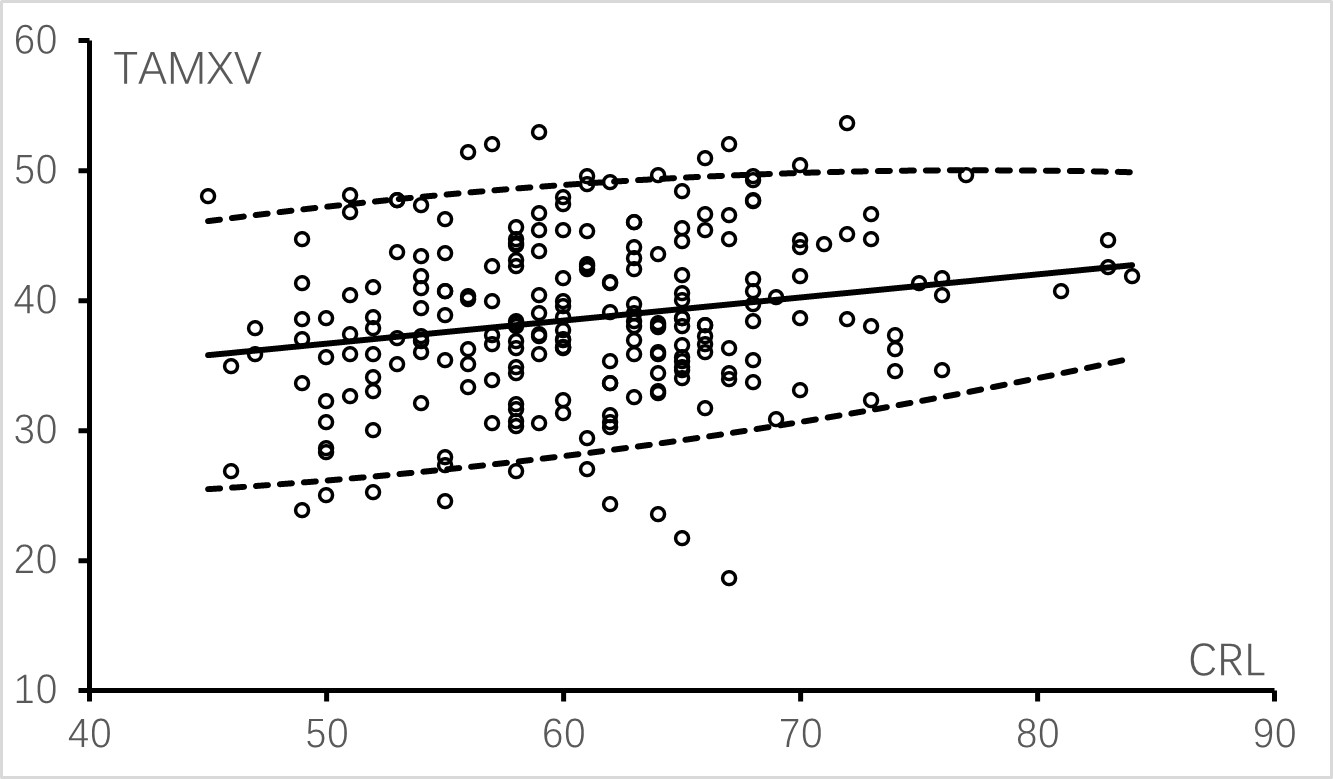

Supplement: S4 Fig — (TIF) [file pone.0312874.s004.tif]
