# Supplementary material for: Reference values of normal fetal ductus venosus Doppler flow measurements at 11–14 weeks of gestation
Source: PLoS One. 2024 Oct 28;19(10):e0312874. doi: 10.1371/journal.pone.0312874 (PMC11516001; doi:10.1371/journal.pone.0312874)
Supplement: Attachment — Submitted filename: Response to Reviewers.docx [file pone.0312874.s006.docx]

Dear Editor, Dear Reviewers,

Thank you very much for taking the time to review this manuscript. I truly appreciate all of your thoughtful comments and suggestions! Please find my detailed responses below, along with my revisions and corrections in the re-submitted files.

**Reviewer #1:**

**The timeframe and setting are clearly stated. However, it would be beneficial to specify the hospital's location for context. Additionally, consider mentioning the number of participants (if available) to give readers a better sense of the study's scale.**

Thank you for your meticulous review and valuable suggestions. We have added the hospital's address into the article. Our study encompassed a total of 343 fetuses; however, due to incomplete follow-up data, some were not included in the article.

**Doppler Ultrasonography (Lines 76-94): This is a crucial part of the methodology. It might be helpful to specify whether the angle of <30° is based on specific guidelines or studies.**

The Certification training section on Ductus venosus flow on the British Fetal Medicine Foundation (FMF) training website states that "The insonation angle should be less than 30 degrees." （https://fetalmedicine.org/fmf-certification-2/ductus-venosus-flow）We have taken this criterion into consideration.

**Additionally, the process of repeating measurements and taking the mean value is good practice, but it could be elaborated on to explain why three repetitions were chosen.**

We consulted existing literature and calculated the average of three measurements to enhance accuracy. Considering that conducting measurements only once or twice may lead to greater errors, while excessive measurements could extend examination duration, we opted for averaging three measurements in accordance with the ALARA (As Low As Reasonably Achievable) principle for fetal ultrasound examinations. This approach aims to achieve optimal measurement precision within a minimized examination timeframe

**Lines 113-120 (Study Population): This is a clear and concise summary of the final study population. The inclusion of demographic data (mean maternal age, BMI, etc.) is useful. However, it might be helpful to specify what "median gestation and delivery numbers" refer to—whether it is the** **number of pregnancies and deliveries or another metric.**

Yes, this refers to the median number of pregnancies and deliveries. Thank you for your correction. The expression has been modified in the text.

**Lines 122-124 (DV PI Results): The results are presented clearly, and the statistical significance is noted. However, the sentence is somewhat dense. It may be helpful to split it: "For the 224 normal fetuses, the DV PI P5 and P95 ranged from 1.0007 and 1.3415 for a CRL of 45 mm to 0.9734 and 1.2115 for a CRL of 84 mm. This correlation with CRL values was statistically significant (Pearson's r = -0.163; P = 0.015).**

Thank you for your modification suggestions. We have implemented the changes as per your advice in the main text to render the sentence more succinct and lucid.

**Weaknesses:**

**Sample Size: The study might benefit from a larger sample size to enhance the statistical power and generalizability of the findings.**

**Single-Center Study: The research appears to be conducted at a single center. Multi-center studies could provide more diverse data and validate the results across different populations.**

The single-center design and the relatively small sample size are indeed the deficiencies of this study. We are highly grateful to the reviewer for highlighting this issue. Please grant us an opportunity to elaborate on these limitations. The single-center study was conducted as we aimed for better quality control, to minimize measurement errors introduced by different measurement techniques among different hospitals and personnel, and to ensure accurate follow-up results. Thus, we initiated our research with a single-center study as the first step. Our sample size was relatively limited; however, during the research process, we observed some abnormal cases and discovered that an increase in DVPI might be associated with adverse pregnancy outcomes. We aspire to publish an article on the normal reference range of DVPI to assist more physicians in providing clinical consultation and guidance for pregnant women. Due to the relatively small number of abnormal cases discovered and the fact that we are still in the process of accumulating cases, abnormal cases are not discussed in this paper. We will undertake multi-center studies with a larger sample size in the subsequent process.

**Limited Gestational Range: The study focuses on the 11–14 weeks of gestation period. Expanding the gestational range could provide a more comprehensive understanding of fetal ductus venosus flow dynamics.**

Further research on DVPI across various gestational weeks is certainly important. However, our study specifically concentrates on the early pregnancy screening period. The ISUOG guidelines recommend that qualitative assessment of DV be routinely performed during this stage, as it plays a vital role in aneuploidy screening. Additionally, the latest ISUOG guidelines suggest that elevated DVPI may correlate with increased detection rates of aneuploidy. Consequently, we examined the normal values of DVPI at 11-14 weeks. Abnormalities in DVPI during mid and late pregnancy could have unique clinical implications, such as fetal growth restriction, which also deserves attention; however, this topic falls outside the current paper's scope. We sincerely appreciate the reviewers for providing us with valuable suggestions for future research directions. Multi-center studies, larger sample sizes, and investigations into normal values of DVPI during mid and late pregnancy are potential avenues we look forward to exploring.

**Reviewer #2: well written provide quantitative assessment in addition to qualitative. Though qualitative assessment of Ductus Venosus flow on its own is a pretty good marker to add up to the existing aneuploidy screening tools.
My Question: How much weightage would you give to addition of quantification of ductus in addition to the overall screening methods?**

Thank you for raising such an insightful question. We took the time to review the fetuses that were excluded from our study due to structural malformations or chromosomal abnormalities. Among these, we identified five cases with normal NT and a positive DV a wave, yet they exhibited abnormally elevated PI values. Specifically, one case was confirmed as trisomy 21 through amniocentesis, another presented with a diaphragmatic hernia along with increased echogenicity of both kidneys, one had an atrioventricular septal defect also confirmed as trisomy 21 by amniocentesis, one showed a single umbilical artery, and another unfortunately experienced intrauterine demise at 16 weeks of gestation. Therefore, we believe that the increase in DVPI during early pregnancy is not only linked to an elevated risk of aneuploidy but may also be associated with adverse pregnancy outcomes like structural anomalies. However, given the limited sample size, we did not delve into discussing the cases of abnormally high DVPI and their implications within this article; instead, we focused on presenting the normal values of DVPI for reference by other researchers. We are actively continuing to accumulate more cases and look forward to uncovering additional insights regarding quantitative assessment of DV in future studies.

We sincerely appreciate the hard work of the Editors and Reviewers, and we hope that our corrections will be well received. Once again, thank you very much for your valuable comments and suggestions.

Sincerely,

Xiangyi Dong

dongxy907@163.com
